# Collaborative Multiscale and Wavelet-Based Fusion Network for Leakage Area Semantic Segmentation of Ultrawide Field Fluorescein Angiography

**DOI:** 10.1167/tvst.15.3.22

**Published:** 2026-03-24

**Authors:** Hongzhe Han, Huilin Liang, Dan Cao, Zijun Lei, Shi Tang, Hanyu Guo, Qianjin Feng

**Affiliations:** 1 School of Biomedical Engineering, Southern Medical University, Guangzhou, Guangdong, China; 2 Guangdong Cardiovascular Institute, Guangdong Provincial People's Hospital, Guangdong Academy of Medical Sciences, Guangzhou, Guangdong, China; 3 Guangdong Eye Institute, Department of Ophthalmology, Guangdong Provincial People's Hospital (Guangdong Academy of Medical Sciences), Southern Medical University, Guangzhou, Guangdong, China; 4 Guangdong Province Engineering Laboratory for Medical Imaging and Diagnostic Technology, Southern Medical University, Guangzhou, Guangdong, China

**Keywords:** UWFA, leakage, segmentation

## Abstract

**Purpose:**

Diabetic retinopathy (DR) is a serious ocular complication of diabetes, affecting about 30%–40% of patients. It primarily damages the retinal microvascular system and can result in visual impairment or even blindness. A hallmark lesion of DR is vascular leakage, which is typically observed in the late-phase images of ultra-widefield fluorescein angiography (UWFA). However, accurately segmenting leakage regions in UWFA remains a challenge because of their irregular and heterogeneous morphology, as well as the substantial computational demands associated with the high-resolution nature of UWFA images.

**Methods:**

We propose a deep learning framework that combines multiscale sampling with two-dimensional wavelet transforms and an exponential moving average (EMA) mechanism to fuse global and local features. A cross-guided neighborhood refinement strategy is further introduced to enhance boundary accuracy.

**Results:**

The experimental results demonstrate that (1) the model exhibits an optimal performance when the EMA parameter is set to 0.3; (2) the performance of the UNet-Wavelet network significantly surpasses traditional networks; and (3) using a multiscale fusion framework confers greater robustness compared with non-framework approaches.

**Conclusions:**

We validated our method on a UWFA dataset from Guangdong Provincial People's Hospital and Foshan Second People's Hospital, and the results demonstrated that our model achieves efficient and accurate segmentation of leakage regions in UWFA images.

**Translational Relevance:**

By addressing the irregular morphology and high-resolution complexity of UWFA images, our method enhances segmentation accuracy and computational efficiency, enabling more objective and timely clinical quantification of DR-related leakage and potentially supporting earlier intervention for affected patients.

## Introduction

Diabetic retinopathy (DR) is a common small-vessel complication of diabetes, affecting about 30%–40% of patients.^1^^,^^2^ It leads to gradual damage of retinal vessels and can cause visual loss or even blindness. Clinically, DR is classified as nonproliferative (NPDR) or proliferative (PDR). NPDR is characterized by microaneurysms, hemorrhages, and hard or soft exudates, whereas PDR is defined by abnormal new vessels on the retinal surface, which may lead to vitreous hemorrhage and tractional retinal detachment.

Clinically, multiple diagnostic modalities are used to evaluate DR and its impact on vision. Routine visual acuity testing offers a preliminary assessment of visual function, while fundus examinations, typically performed through ophthalmoscopy after pupil dilation, provide direct visualization of retinal morphological changes. Optical coherence tomography (OCT),^3^ a modern, noninvasive imaging technique, generates high-resolution cross-sectional images of the retinal microstructure and is particularly valuable for detecting and monitoring macular edema.

Fluorescein fundus angiography (FFA) remains a key imaging technique for DR evaluation. In this procedure, fluorescein sodium is administered intravenously, and, under light excitation at specific wavelengths, the retinal vasculature is visualized in detail. This allows for precise assessment of vascular permeability, perfusion status, and vascular lesions, enabling clinicians to identify MA, hemorrhages, leakage regions, and neovascularization. Ultrawide field fluorescein angiography (UWFA) extends the capabilities of traditional FFA by providing a panoramic view of the retina, thereby facilitating the detection of peripheral lesions that may otherwise be overlooked.^4^ Owing to its broader coverage, UWFA holds significant clinical value in the early identification of widespread peripheral retinal pathology and in guiding treatment planning.

In FFA, fluorescein sodium circulates to the fundus, and on excitation by specific wavelengths, the dye within retinal vessels emits fluorescence. Leakage from abnormal vessels produces distinct hyperfluorescent patterns, which serve as indicators of pathological changes. These leakage regions appear as bright areas, where fluorescence intensity often increases progressively over time. Although FFA enables detailed visualization of vascular structures, it requires intravenous administration of fluorescein dye, which may lead to adverse effects such as severe allergic reactions, arm pain, and, in rare cases, retinal vascular complications.^5^^,^^6^

Clinically, the detection and delineation of leakage areas are commonly performed through manual annotation or threshold-based segmentation methods. Threshold segmentation^7^ operates by setting one or more threshold values to classify image pixels into distinct categories, thereby separating normal and abnormal regions. However, its performance is highly dependent on the choice of threshold, and a single threshold is often insufficient to capture the diverse and complex features present in leakage images. Furthermore, because retinal blood vessels also appear as bright hyperfluorescent structures in FFA images, accurate separation of vessels from leakage areas typically requires extensive manual effort in combination with threshold-based techniques.

In recent years, artificial intelligence (AI)–driven image segmentation technologies have demonstrated notable advantages in the automated identification and quantitative analysis of leakage regions. Compared with traditional methods, AI-based approaches offer superior objectivity, speed, and cost-efficiency.^8^ Nonetheless, segmenting leakage regions in UWFA images remains particularly challenging. These difficulties primarily arise from the following factors:

### Complexity and High Costs of UWFA

As a recently developed fundus imaging technique, UWFA involves sophisticated equipment and intricate operational procedures, which in turn generate substantial research costs. These costs limit the involvement of many research institutions and hinder the collection of sufficient research samples. In addition, accurate annotation of lesions demands advanced ophthalmic expertise, and the process itself is both labor-intensive and time-consuming, requiring trained professionals. As a result, the data preparation stage advances slowly.

### High Computational Demand of Ultrahigh-Resolution Images

Ultra-widefield images belong to the class of ultrahigh-resolution medical images, which place exceptionally heavy demands on computational power. Analyzing such images requires extensive resources to process high-dimensional data, while also imposing strict requirements on both hardware performance and storage capacity. Furthermore, the added detail and inherent noise within these images increase the complexity of feature extraction, making it more difficult for models to learn and generalize with precision.

### Loss of High-Resolution Details and Contextual Information

In semantic segmentation tasks, strategies such as down-sampling or patch-cropping are often applied to ultrahigh-resolution images. However, these approaches may compromise critical high-resolution details and diminish valuable spatial context. For medical imaging, fine details like tissue texture, lesion boundaries, and subtle abnormalities are essential for accurate disease detection and diagnosis. Equally important, spatial context provides information about the relative positions of tissues and organs, which allows clinicians to interpret lesions within their anatomical environment. This contextual understanding supports the assessment of lesion spread, interactions with surrounding tissues, and their relationships with adjacent structures.

### Irregular and Diverse Morphologies of Leakage Areas

Leakage areas often display irregular and highly diverse morphological patterns. In fundus images, these regions may appear blurred or exhibit low contrast, making it challenging to differentiate them from normal tissues.

To address this, further research is needed to achieve high-precision segmentation of non-perfused regions in fundus images using advanced AI techniques. In this study, we propose an intelligent segmentation method for fundus leakage areas that leverages multiscale sampling and two-dimensional discrete wavelet transform (2D DWT) fusion to improve both accuracy and efficiency in UWFA segmentation.

Specifically, we introduce a novel model termed the Collaborative Multiscale and Wavelet Fusion Network (CMWNet), which integrates global full-field images with cropped local regions during both training and inference. The architecture comprises two main branches: large-scale and small-scale, where the large-scale branch processes down-sampled global images, whereas the small-scale branch focuses on local cropped regions. These branches are jointly optimized through an exponential moving average (EMA) mechanism,^21^ inspired by the semi-supervised “mean teacher” framework, where the small-scale branch acts as the teacher model and the large-scale branch as the student model. Through EMA, the two branches exchange weights for collaborative optimization, ensuring the capture of fine-grained details while preserving global contextual integrity, thereby minimizing information loss. To further improve the spatial consistency and boundary integrity of the predictions, we additionally propose a novel post-processing method named *Cross-Guided Neighborhood Refinement* (CGNR), which refines small-scale predictions under the structural guidance of the large-scale output. Both branches adopt a U-Net structure enhanced with wavelet transform, allowing low-frequency components to capture the overall structure while emphasizing texture variations and contrast in leakage regions. This design enhances feature extraction, boosts segmentation precision, and increases robustness. Overall, CMWNet effectively integrates global contextual information with local details, whereas EMA balances their contributions and the wavelet transform highlights critical image features. Experimental results confirm that CMWNet achieves superior performance in ultrahigh-resolution image segmentation and provides a promising solution for future medical image analysis.

Our main contributions are as follows:

We developed a novel CMWNet to address the challenges of ultrahigh-resolution image segmentation in UWFA. By resizing both down-sampled global images and cropped local images to a uniform input size, the model significantly reduces the computational burden on graphics processing units. Furthermore, the integration of large- and small-scale branches with an EMA mechanism for weight sharing enables the network to accurately capture fine-grained medical image details while simultaneously preserving the integrity of global contextual information.

We propose a novel post-processing strategy named Cross-Guided Neighbourhood Refinement, inspired by morphological operations such as erosion and dilation. CGNR leverages the structural guidance of the large-scale model to refine small-scale predictions by selectively updating uncertain pixels based on their neighbourhood consistency. This approach effectively preserves fine details while enhancing spatial continuity, particularly at lesion boundaries.

We propose a U-Net-based framework enhanced with 2D-DWTs (UNet-Wavelet) to address the irregular and diverse morphologies of leakage areas in segmentation tasks. By extracting low-frequency components through wavelet transforms, the model emphasizes global structural details and texture variations, thereby significantly improving recognition accuracy.

To further enhance performance, we integrate CMWNet with UNet-Wavelet, leveraging the benefits of small-scale image cropping and data augmentation to overcome the challenge of limited UWFA datasets. This approach not only increases the diversity of training samples but also strengthens the model's robustness and adaptability, enabling more reliable segmentation results.

## Methods

### Dataset

**Figure 1.**
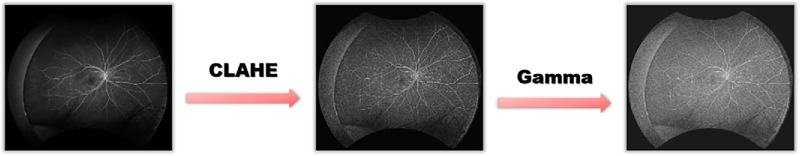
Preprocessing illustration.

This study collected 183 frontal images of late-stage UWFA using an Optos California ultra-widefield retinal camera at Guangdong Provincial People's Hospital between September 2021 and June 2024. Lesion annotations were performed by two trained medical students with the medical imaging software Mimics 23, focusing on leakage areas. Following annotation, the segmented images were compared against the original images and subsequently reviewed by a retinal expert with extensive UWFA image processing experience. Any images with unsatisfactory segmentation were manually corrected under expert supervision. All UWFA images were preprocessed using contrast-limited adaptive histogram equalization^15^ and gamma correction. The preprocessing workflow is illustrated in Figure 1.

### Network

#### Collaborative Multiscale Network

**Figure 2.**
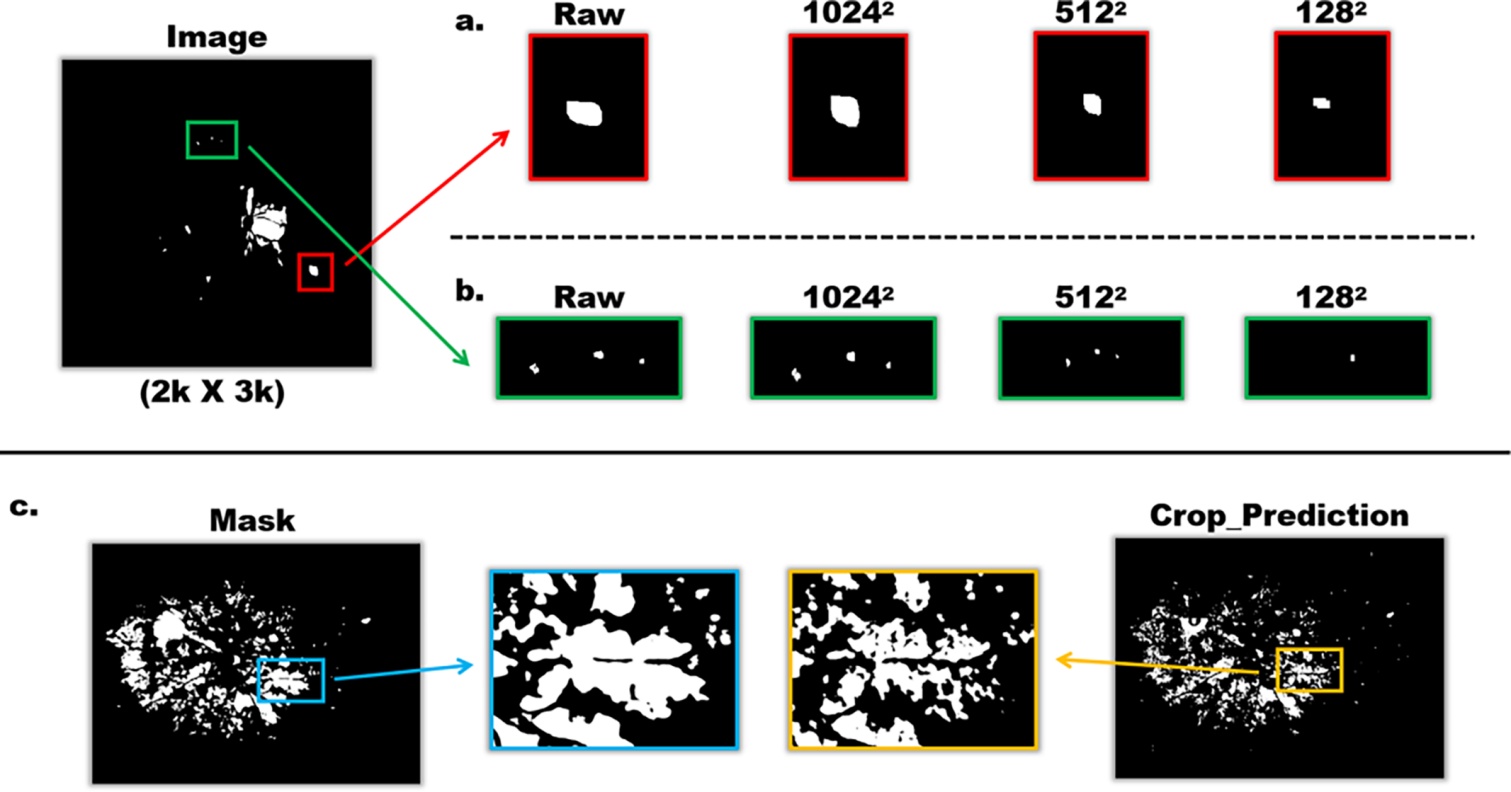
Comparison of Down-sampling and Cropping. (**a**) Loss of fine structures after down-sampling; (**b**) Disappearance of subtle leakage signals after down-sampling; (**c**) Fragmented leakage regions caused by patch-wise cropping.

When addressing training and inference tasks for ultrahigh-resolution images under GPU memory constraints, two common strategies are typically applied: (1) down-sampling the entire image and (2) cropping the global image into smaller patches. Although these approaches partially mitigate hardware limitations, they inevitably introduce losses in image information. Down-sampling alone can eliminate fine structures, as shown in Figure 2a, where the gradual loss of white details reduces segmentation accuracy. Similarly, Figure 2b highlights that subtle leakage information may disappear after down-sampling, a risk that must be avoided. Cropping, on the other hand, introduces a different limitation: the absence of global semantic context. As shown in Figure 2c, compared with the continuous vertical annotations in the uncropped original image, the leakage areas in cropped patches appear fragmented and disconnected. This loss of spatial context and neighborhood dependency hampers accurate discrimination between “lesion” and “non-lesion” regions.

To address the limitations of the aforementioned approaches, we propose an innovative framework that integrates down-sampling and cropping strategies in a complementary manner to optimize ultrahigh-resolution image processing. Down-sampling preserves the global spatial context and adjacency information of the original image, while cropping mitigates the reduction and loss of pixel-level details. By systematically combining these methods, our multiscale collaborative network effectively overcomes the challenges of ultrahigh-resolution image segmentation under memory constraints. This approach maintains the overall structural integrity and global significance of images while simultaneously preserving critical local details.

**Figure 3.**
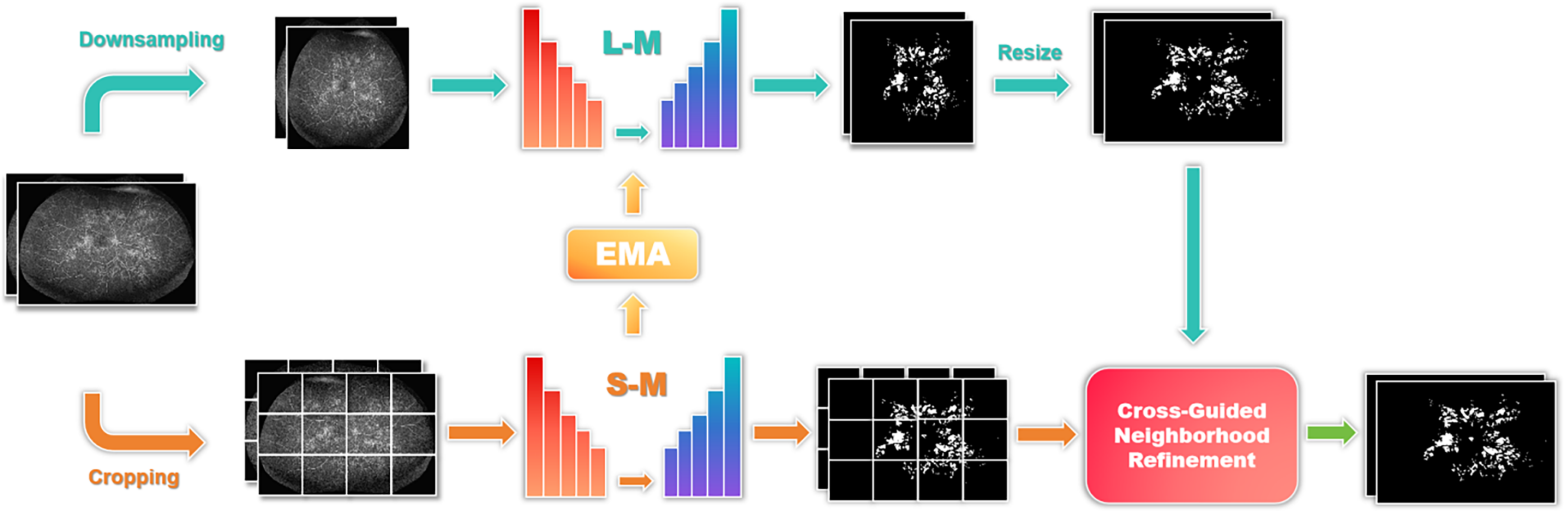
Diagram of the collaborative multiscale network framework.

In Figure 3, we present the architecture of our Collaborative Multiscale Network. The process begins with several preprocessing steps applied to the original FFA super-resolution images, including both down-sampling and cropping, to generate corresponding low-resolution representations. Specifically, given N super-resolution images, each image is down-sampled to produce an equivalent number of low-resolution images, expressed as ${\left\{D_{i}\right\}}_{i\;=\;1}^{N},D_{i}=f_{ds}\left(S_{i}\right),i=1,2,...,N$. Simultaneously, these *N* super-resolution images are cropped into *M* low-resolution images, denoted as ${\left\{C_{j}\right\}}_{j=1}^{M}$, where $C_{j}=f_{crop}\left(S_{i}\right),j=1,2,...,M,i=1,2,...,N$. Here, Sᵢ represents an individual super-resolution image. The down-sampled and cropped images are then input into the large-scale and small-scale models, respectively, both of which are based on the “UNet-Wavelet” architecture that integrates the 2D DWT into the symmetric U-Net encoder-decoder structure. The small-scale model further employs an EMA mechanism to update the parameters of the large-scale model, with *α* serving as the EMA adjustment coefficient.

The model output is optimized through a weighted combination of losses. Here, *L_1_* represents the loss from the large-scale model and *L_2_* represents the loss from the small-scale model, both calculated as weighted sums of binary cross-entropy loss, Dice loss,^9^ and Tversky loss.^10^ A joint loss, *L_3_*, is defined as *L*_3_ = (1 − α)*L*_1_ + α*L*_2_, where *α* is the EMA adjustment parameter. In addition, L₄ represents the consistency loss between the outputs of the large- and small-scale models. The final combined objective is given as *Loss* = *L_3_* + *βL_4_*, where *β* is a random coefficient ranging between 0 and 1. This composite loss function enhances both predictive accuracy and robustness.

The final output is generated by up-sampling the predictions from the large-scale model to match the original image dimensions, denoted as ${\left\{{\hat{Y}}_{i}^{G}\right\}}_{i=1}^{N}$, where ${\hat{Y}}_{i}^{G}=f_{us}\left(GM(D_{i})\right)$,i=1,2,...,N. Similarly, predictions from the small-scale model are sequentially reassembled (re-montaged) to the original size, represented as $\left\{{\hat{Y}}_{j}^{L}\right\}{}_{j=1}^{N}$, where ${\hat{Y}}_{j}^{L}=f_{montage}\left(LM(C_{j})\right)$,j=1,2,...,M. These two sets of predictions are then fused through a weighted combination to produce the final segmentation labels.

**Figure 4.**
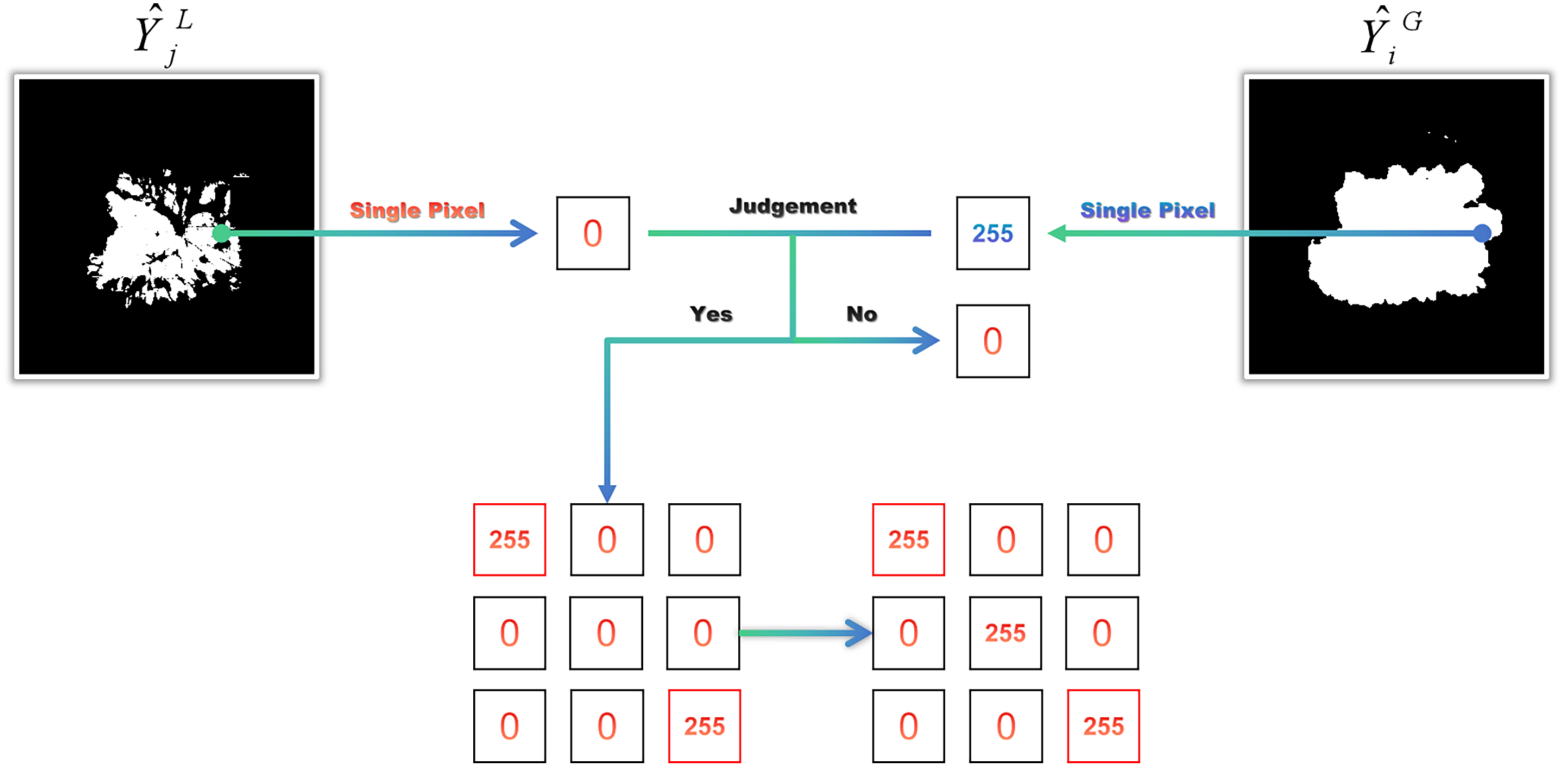
Cross-guided neighborhood refinement.

To further refine prediction quality, a Cross-Guided Neighborhood Refinement strategy is applied (Fig. 4). In this step, ${\hat{Y}}_{j}^{L}$ is treated as the base mask to be updated under the guidance of ${\hat{Y}}_{i}^{G}$. Specifically, for each pixel in ${\hat{Y}}_{j}^{L}$ with a value of 0, the corresponding pixel in ${\hat{Y}}_{i}^{G}$ is examined. If the pixel value in ${\hat{Y}}_{i}^{G}$ equals 255, it is marked as a candidate for update. The eight-connected neighborhood of the candidate pixel in ${\hat{Y}}_{j}^{L}$ is then evaluated: If any neighboring pixel has a value of 255, the central pixel is updated to 255; otherwise, it remains 0. This procedure ensures that only those foreground regions in ${\hat{Y}}_{i}^{G}$ that are spatially coherent with existing structures in ${\hat{Y}}_{j}^{L}$ are retained, thereby enhancing boundary continuity while suppressing isolated false positives.

#### UNet-Wavelet

**Figure 5.**
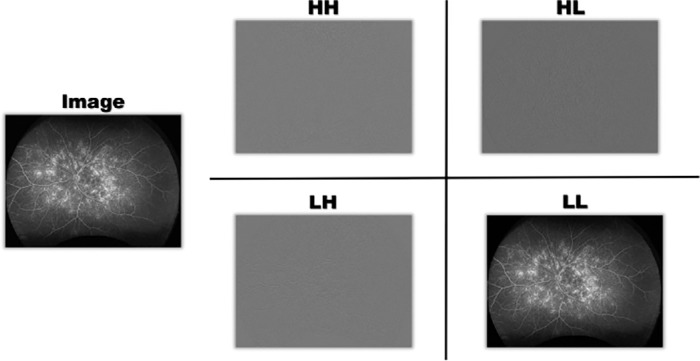
Wavelet decomposition diagram.

With the advent of advanced segmentation modules such as dilated convolutions,^11^ feature pyramids,^12^ and attention mechanisms,^13^ convolutional neural networks have demonstrated both robustness and effectiveness in extracting complex image features. To further improve segmentation performance, we leverage the characteristics of segmented images to guide network architecture design, thereby enhancing adaptability to UWFA-specific image features. In particular, we observe that leakage regions exhibit higher sensitivity to wavelet transforms. The low-frequency components obtained from the wavelet transform capture global structures and salient features, while emphasizing details and texture variations that enhance the contrast and visibility of leakage regions. This significantly improves both recognition accuracy and robustness. As illustrated in Figure 5, wavelet decomposition of the original image produces four components: low-frequency (LL), horizontal high-frequency (LH), vertical high-frequency (HL), and diagonal high-frequency (HH). Leakage regions are less visible in the high-frequency components (HH, HL, and LH) due to blurred boundaries lacking clear contours. Based on this observation, we designed the UNet-Wavelet network, which incorporates the 2D DWT.

In our implementation, we adopt the Haar wavelet as the mother wavelet owing to its simplicity and efficiency in capturing edge and texture information. Wavelet decomposition is performed at four levels, which provides an optimal trade-off between preserving spatial resolution and extracting multiscale contextual information.

**Figure 6.**
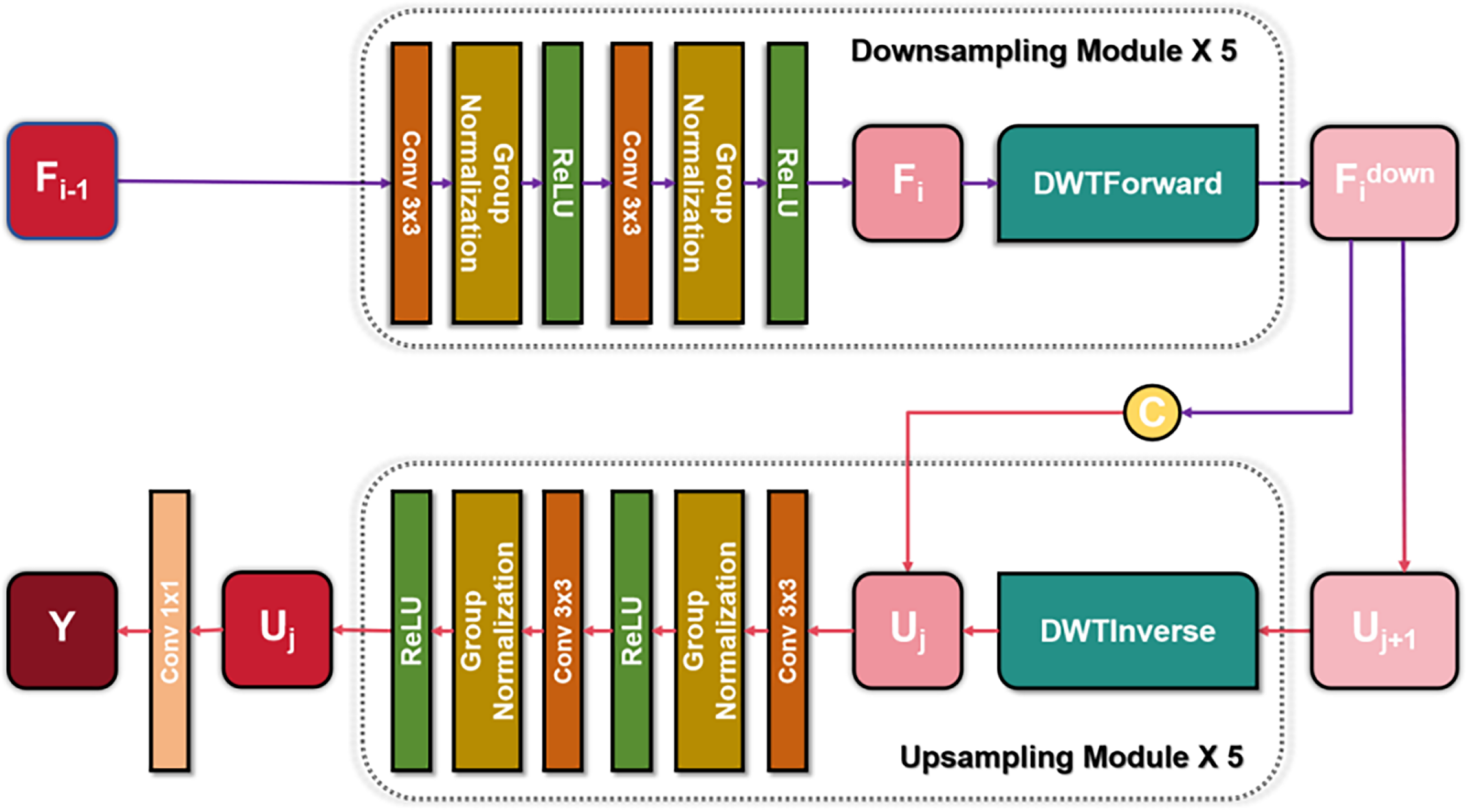
UNet-wavelet network framework.

The proposed network architecture follows the symmetric encoder-decoder structure of U-Net^14^ (Fig. 6). The down-sampling path extracts spatial features from the input image, whereas the up-sampling path reconstructs a dense segmentation map. Each down-sampling module begins with two 3 × 3 convolution operations, followed by group normalization and ReLU activation, and then applies a forward wavelet transform (DWTForward) instead of conventional max pooling. This replacement reduces spatial resolution while retaining low-frequency information, thereby enabling the extraction of multiscale features. The process is iterated five times, progressively reducing spatial dimensions and doubling the number of feature channels to capture increasingly abstract features:

$$\begin{array}{ccc} & & \;F_{i}=ReLU(GroupNorm(Conv_{3\times 3}\\ & & \;(ReLU\left(GroupNorm\left(Conv_{3\times 3}\left(F_{i-1}\right)\right)\right)))) \end{array}$$

$$\begin{array}{c} F_{i}^{down}=DWTForward\left(F_{i}\right) \end{array}$$

Here, *i* denotes each down-sampling module, *F_i_* represents the feature map within the *i^th^* down-sampling module, and $F_{i}^{down}$ is the down-sampled feature map.

Subsequently, the down-sampling path is integrated with the up-sampling path through stored multiscale features, which are concatenated to preserve spatial information. The up-sampling path consists of inverse wavelet transform (DWTInverse) operations and convolutional modules. In each module, up-sampling is performed using DWTInverse to increase the feature map resolution, followed by two 3 × 3 convolution layers to reintegrate and refine feature representations. Additionally, feature maps from the corresponding down-sampling stages are incorporated via skip connections, ensuring the recovery of fine details and maintaining consistency between high-level and low-level features:

$$\begin{array}{c} U_{j}=DWTInverse\left(U_{j+1}\right) \end{array}$$

$$\begin{array}{c} U_{j}=U_{j}+F_{j}^{down} \end{array}$$

$$\begin{array}{ccc} & & \;U_{j}=ReLU(GroupNorm(Conv_{3\times 3}\\ & & \;(ReLU\left(GroupNorm\left(Conv_{3\times 3}\left(U_{j}\right)\right)\right)))) \end{array}$$

In this context, *j* denotes the *j^th^* up-sampling module, and *U_j_* represents the feature map within that module. A 1 × 1 convolution layer is appended after the final up-sampling module to generate the required number of output channels, producing the predicted result: *Y* = *Conv*_1  ×  1_(*U*_1_).

The down-sampling and up-sampling paths are constructed using structurally similar blocks, each consisting of two 3 × 3 convolution layers and either a DWTForward or DWTInverse operation. In the down-sampling path, wavelet transforms are used to reduce spatial resolution while enhancing feature abstraction by doubling the number of feature channels. Conversely, the up-sampling blocks progressively restore spatial resolution using DWTInverse, with convolutional modules reconstructing and refining feature representations. Skip connections further link corresponding down- and up-sampling blocks to facilitate multiscale feature transmission. During up-sampling, these skip connections directly add feature maps from the down-sampling path (after convolution) to the feature maps generated by DWTInverse in the up-sampling path. Feature dimensions are aligned via interpolation, enabling effective feature fusion and accurate reconstruction.

### Data Analysis

To evaluate the performance of the segmentation model, we use the Dice similarity coefficient,^16^ which incorporates both precision (PR) and recall (RC). The metric is computed using the four fundamental classification outcomes: true positive (TP), true negative (TN), false positive (FP), and false negative (FN).

We define PR as:

$$\begin{array}{c} PR=\frac{TP}{TP+FP} \end{array}$$

and RC as the TP Rate (TPR):

$$\begin{array}{c} RC=TPR=\frac{TP}{TP+FN} \end{array}$$

The Dice similarity coefficient is defined by the following equation:

$$\begin{array}{c} DSC=\frac{2\;\times TP}{2\;\times TP+FP+FN} \end{array}$$

Additionally, the 95% Hausdorff distance (HD95) and the average surface distance (ASD) are key statistical measures for evaluating spatial data distributions and are widely used in spatial analysis and medical image segmentation. Specifically, HD95 measures the 95th percentile of the distances from boundary points of one segmentation result to the closest boundary points of the reference segmentation, thereby mitigating the influence of outliers:

$$\begin{array}{c} \;HD_{95}=P_{95}\left(d\left(x_{i},x_{0}\right)\right) \end{array}$$

where *d*(*x_i_*,*x*_0_) is the distance from sample point *x_i_* to the central point *x*_0_, and *P*_95_ denotes the 95th percentile.

ASD focuses on the average distance to the nearest neighbor within the dataset:

$$\begin{array}{c} ASD=\frac{1}{N}\sum_{i=1}^{N}\underset{j\neq i}{min}\left(d\left(x_{i},x_{j}\right)\right) \end{array}$$

where *N* is the total number of data points, and *d*(*x_i_*,*x_j_*) is the distance between sample points *x_i_* and *x_j_*.

### Implementation Details

Before conducting the experiments, the internal dataset was randomly partitioned into training, validation, and test sets containing 111, 29, and 43 images, respectively. In addition, to independently assess model generalizability across clinical centers, we collected an external test set comprising 43 images from a tertiary referral hospital in Foshan, China, which was used exclusively for independent validation. To further ensure robust assessment and reduce bias caused by random splits, we adopted a fivefold cross-validation strategy on the internal dataset. To enhance model generalization and mitigate overfitting, data augmentation was systematically applied. First, the original images were resized using nearest-neighbor interpolation so that both height and width were exact multiples of 512 pixels. Each resized image was then divided into non-overlapping 512 × 512 patches in a grid-like fashion. Finally, augmentation techniques, including random horizontal flipping and color jittering (adjustments to brightness, contrast, saturation, and hue), were applied to the patches to enrich the dataset and improve robustness.

For data loading, we used the PyTorch DataLoader. The training loader shuffled samples randomly with a batch size of four, whereas the test loader preserved sequential order with a batch size of one. Model optimization was performed using the AdamW optimizer with a learning rate of 0.005, *β* parameters of 0.9 and 0.999, a weight decay of 0.00003, and AMSGrad disabled. To adaptively adjust the learning rate, we adopted a cosine annealing scheduler with T_max set to 50 cycles, corresponding to one complete cosine period, and a minimum learning rate of 0.00001.

## Results

### Comparison of Different Values of the EMA Coefficient *α*

In our study, the small-scale model updates the parameters of the large-scale model using an EMA strategy, where *α* serves as the EMA adjustment coefficient. To examine its effect on model performance, we conducted experiments with different *α* values (Table 1). Intuitively, *α* controls the balance between the influence of the small-scale model's current parameters and the historical parameters of the large-scale model: a higher *α* assigns greater weight to the current updates, enabling faster adaptation but risking instability, whereas a lower *α* emphasizes accumulated knowledge, ensuring stability but potentially slowing convergence. As shown in Table 1, performance differences across *α* values are relatively small; however, the model achieves the best overall performance when *α* = 0.3. This suggests that *α* = 0.3 provides an optimal tradeoff, allowing the small-scale model to guide updates effectively while preserving the global representations captured by the large-scale model. This outcome may be attributed to the fact that the large-scale model's global features are more conducive to overall training, producing predicted images with global connectivity more closely aligned with the ground truth. Therefore *α* = 0.3 is adopted for all subsequent experiments.

**Table 1.** Comparison of Performance With Different *α* for Segmentation and Identification of Retinal Leakage Areas on the Test Set

*α*	DCS	HD95	ASD
*α* = 0.1	0.8567	37.2387	9.0541
*α* = 0.3	**0.8582**	**33.0984**	**7.9451**
*α* = 0.5	0.8492	50.0327	9.1005
*α* = 0.7	0.8366	48.8912	10.0068
*α* = 0.9	0.8443	42.1569	9.9510

Bold values indicate the best performance among different *α* settings.

### Comparison With Common Models

**Figure 7.**
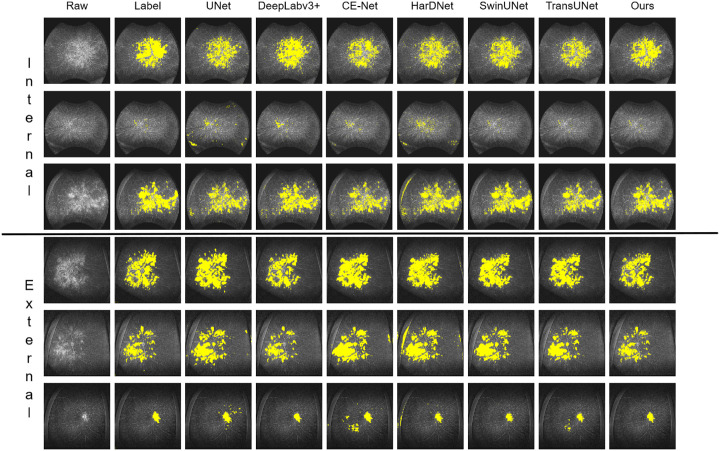
Comparison of results with those of commonly used models.

In diverse application scenarios, we compared the proposed UNet-Wavelet network with several widely used segmentation models, including UNet, Deeplabv3+, CE-Net, and HarDNet, as well as more advanced Transformer-based architectures such as TransUNet and SwinUNet. UNet is a classical encoder-decoder architecture that has become a standard baseline in biomedical image segmentation. Deeplabv3+ incorporates atrous spatial pyramid pooling together with a decoder module to capture multiscale contextual information while preserving spatial details. CE-Net enhances feature representation by integrating a context extractor module and dense atrous convolutions, whereas HarDNet leverages harmonically dense connections to achieve high segmentation accuracy with reduced computational cost. For a fair comparison, the overall experimental setup and architectural configurations were kept unchanged, with only the original UNet-Wavelet network being replaced by these alternative models. All methods were evaluated on both an internal dataset and an independent external dataset to comprehensively assess robustness and generalizability. The comparative results are summarized in Table 2 and illustrated in Figure 7.

**Table 2.** Performance Comparison of Different Models in Segmenting and Identifying Fundus Leakage Regions in the Test Set

Model	Data	DCS	HD95	ASD
Unet	Internal	0.7562	69.6572	25.5643
	External	0.7452	91.1917	26.4464
Deeplabv3+	Internal	0.7528	58.7633	22.0357
	External	0.7697	87.2788	15.7663
CE-Net	Internal	0.7498	44.4861	18.1592
	External	0.7280	122.7067	57.7873
HarDNet	Internal	0.7209	98.8912	54.6875
	External	0.7261	243.6733	76.5197
SwinUNet	Internal	0.8236	36.6795	10.4763
	External	0.7913	**44.5881**	14.7302
TransUNet	Internal	0.8095	40.6464	11.1658
	External	0.7702	56.4508	15.8719
Ours	Internal	**0.8582**	**33.0984**	**7.9451**
	External	**0.8458**	55.5972	**10.1148**

Bold values indicate the best performance for each metric on the internal or external dataset, respectively.

As illustrated in the figure, the UNet-Wavelet network demonstrates markedly superior performance compared to traditional models. This improvement is primarily due to its effective and comprehensive exploitation of leakage region characteristics in FFA. In particular, the wavelet transform emphasizes low-frequency components within images, which include energy-concentrated regions and global structural patterns. These features are critical for accurately identifying leakage areas while simultaneously reducing the influence of noise. By applying wavelet transforms at each encoder layer, our network consistently captures these low-frequency features and integrates them with the original image features, thereby achieving enhanced pixel-level segmentation accuracy.

### Ablation Study on the EMA Mechanism

To assess the contribution of the EMA-based parameter update strategy in CMWNet, we conducted an ablation study by comparing the full CMWNet with two baseline variants in which EMA is disabled: (1) a down-sampling-only network (“Down-Sampling”) and (2) a cropping-only network (“Crop”). In these baseline settings, the down-sampling and cropping branches function as independent sub-models without EMA-driven parameter interaction, whereas in CMWNet the EMA mechanism explicitly couples the large-scale (Down-Sampling) and small-scale (Crop) branches.

**Figure 8.**
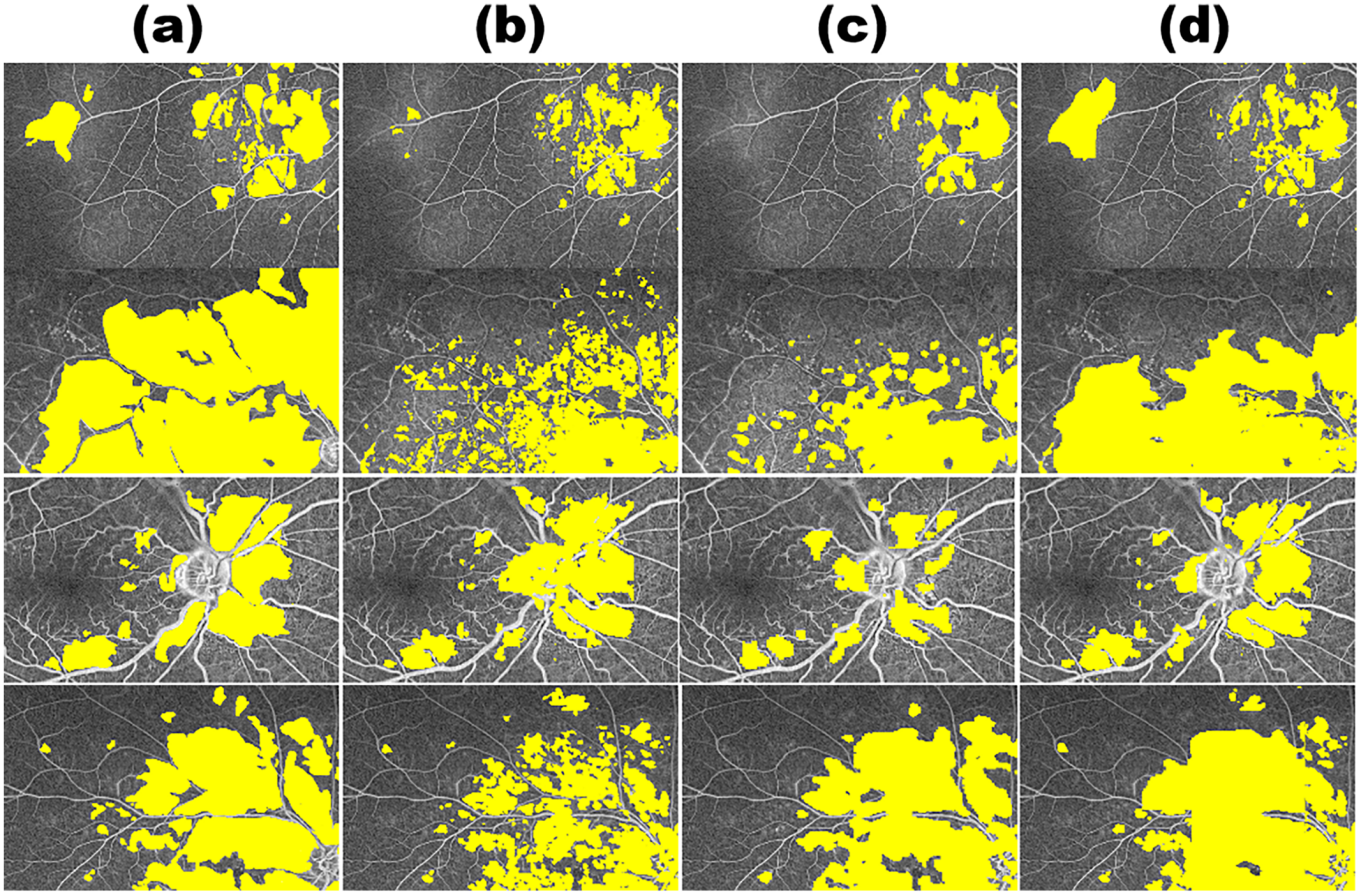
Comparison of (**a**) mask; (**b**) cropping only; (**c**) down-sampling only; (**d**) CMWNet.

Table 3 presents the quantitative results, showing that CMWNet consistently outperforms either baseline when used in isolation. To provide a more intuitive understanding, Figure 8 further shows side-by-side qualitative examples of predictions with EMA enabled (CMWNet) versus EMA disabled (Down-Sampling and Crop). These visual comparisons illustrate that, through EMA-driven knowledge transfer, the small-scale model continuously updates the large-scale model, enabling the network to retain global spatial context while simultaneously refining fine-grained details. When EMA is removed and the two branches are trained independently, the large-scale model tends to miss subtle local structures, whereas the small-scale model often loses spatial contextual continuity.

**Table 3.** Performance Comparison of Retinal Leakage Region Segmentation and Identification in the Test Set With and Without the Use of the EMA Mechanism

Method	DCS	HD95	ASD
Crop	0.8385	40.3721	9.5814
Down-Sampling	0.7702	63.8056	18.9267
CMWNet	**0.8582**	**33.0984**	**7.9451**

Taken together, these findings indicate that the EMA mechanism is not a peripheral enhancement but a core component for maximizing cross-scale synergy. Its removal leads to a clear decline in both regional accuracy and boundary precision, underscoring its necessity for reliable fundus leakage segmentation.

## Discussion

The development and validation of deep learning algorithms in the medical field have progressed rapidly, driven by the need for swift clinical integration to address the increasing burden of exponentially growing patient appointments in healthcare institutions. Although many of these algorithms are designed for conditions already well understood by clinicians, they provide crucial support in addressing challenges associated with the accurate and consistent measurement of pathological outcomes, even when time constraints are not a factor. Their primary strengths lie in automation and efficiency, enabling the processing of large volumes of complex data and thereby improving diagnostic consistency and accuracy. Moreover, these algorithms can identify subtle patterns that may escape human observation, enhancing the potential for early detection and preventive intervention. Nonetheless, continuous cross-validation and extensive clinical evaluation remain essential to ensure their reliability and clinical utility. Ultimately, the advancement of such technologies not only strengthens diagnostic precision but also contributes significantly to improving the overall efficiency of healthcare systems.

A critical challenge in the development of deep learning models for medical imaging is the limited availability of large-scale datasets, largely due to the difficulty of data acquisition and the need for expert annotation. Small datasets increase the risk of overfitting, where models tend to memorize training examples rather than learning generalizable representations. To address this issue, this study uses a fivefold cross-validation strategy, ensuring that each data sample contributes to both training and validation across multiple iterations. This approach enhances model robustness, improves generalization capability, and provides a more reliable evaluation of performance.

Another important challenge arises from domain shift, which occurs because of variations in imaging systems or institutional protocols. For example, UWFA images obtained from devices such as Optos California and Heidelberg differ substantially in brightness, field distortion, and resolution. Because the current model is trained exclusively on images from a single device, its generalizability to external datasets remains uncertain. Such domain-specific biases may limit performance when applied in diverse clinical settings. Future work should therefore prioritize external validation using datasets acquired from different imaging platforms or institutions. Additionally, the integration of domain adaptation techniques (such as adversarial learning, feature alignment, or image normalization) could further improve the model's robustness and adaptability across heterogeneous data sources.

Super-resolution image segmentation seeks to enhance both the spatial resolution and semantic understanding of low-resolution images by integrating advanced computer vision techniques with deep learning models. This approach combines super-resolution reconstruction with refined segmentation strategies, thereby optimizing feature extraction and multiscale information fusion. As a result, it improves target detection and region delineation while preserving fine edge details. Owing to these advantages, the technology has found broad application in fields such as medical image analysis, remote sensing, and video surveillance, where high-precision visual interpretation is essential. A number of super-resolution image segmentation methods have been proposed in recent years. For example, GLNet^17^ introduces a collaborative global-local framework, leveraging contextual information from the global branch while refining details through the local branch to enhance segmentation accuracy. Building on this, the Patch Proposal Network (PPN)^18^ incorporates a classification branch to identify key local patches and integrate them into the global image, thereby improving segmentation efficiency. ISDNet,^19^ on the other hand, achieves ultrahigh-resolution image segmentation through a bilateral architecture that combines deep and shallow networks. In this study, we extend the GLNet framework by introducing an EMA mechanism to adaptively adjust segmentation weights between large- and small-scale images. This adjustment reduces detail loss in large-scale (down-sampled) images while compensating for the lack of spatial continuity in small-scale (cropped) images. Unlike conventional weight-sharing strategies, EMA provides a more deterministic and stable means of knowledge transfer between scales. Such refinement is particularly crucial in medical image segmentation, where accurate delineation of small lesion areas is often more important than global segmentation. Annotating these subtle regions typically requires considerable time and effort from expert clinicians and carries a high risk of omission. The integration of AI can help mitigate these challenges by reducing annotation time while improving segmentation accuracy and reliability.

Although our approach is conceptually inspired by prior works such as GLNet, PPN, and ISDNet, it introduces key innovations that distinguish it from these frameworks. Unlike GLNet and PPN, which rely on global-local fusion or patch-proposal mechanisms, CMWNet employs an EMA strategy to dynamically align the learning of small-scale (local) and large-scale (global) branches. This design improves memory efficiency by avoiding redundant forward passes and enforces consistent segmentation performance across scales. In contrast to ISDNet, which separates shallow and deep layers through a bilateral framework, CMWNet's EMA-guided collaborative fusion better preserves spatial continuity and demonstrates superior capability in capturing the irregular and heterogeneous morphologies of leakage regions in UWFA.

UWFA provides an expansive view of the fundus, enabling detailed visualization of both choroidal and retinal vascular networks. However, the segmentation of leakage regions remains challenging. First, leakage areas often display irregular, diffuse, and dynamically changing patterns, which complicate accurate automated delineation. Second, the wide field of imaging introduces geometric distortion and uneven brightness, impairing edge detection and boundary precision. Furthermore, physiological variations and image noise exacerbate the difficulty of distinguishing pathological leakage from normal structures. To address these challenges, Jiang et al.^20^ proposed a macula-centered method that used concentric circles for regional analysis with golden annotations for key areas, while applying vascular filtering and intensity-gradient normalization to segment leakage regions. Although effective in some contexts, this method presents two notable limitations: (i) it requires prior vascular segmentation in UWFA images, and (ii) performance is sensitive to inter-device differences in UWFA intensity gradients, making consistent segmentation difficult across datasets and augmentation strategies. To overcome these limitations, this study introduces a network architecture that integrates the symmetric encoder-decoder design of U-Net with 2D-DWT. The incorporation of wavelet decomposition enables effective extraction of low-frequency information, thereby enhancing image detail, texture variation, and contrast. This not only improves the visibility and salience of leakage regions but also supports robust one-time automatic segmentation without the need for auxiliary vascular preprocessing or device-specific adaptations.

To address the challenges of training and inference with ultrahigh-resolution UWFA images under GPU memory constraints, conventional strategies such as down-sampling and cropping are insufficient, as they often cause detail loss and compromise semantic integrity. To overcome these limitations, we developed CMWNet, an innovative framework that integrates both down-sampling and cropping to optimize high-resolution image processing. Specifically, down-sampling is employed to preserve global spatial context, while cropping is used to retain fine-grained pixel-level details. Coupled with an EMA mechanism and a multi-weight loss strategy, CMWNet achieves robust integration of global and local information, thereby enhancing prediction accuracy and overall segmentation reliability. In parallel, we designed a UNet-Wavelet architecture that replaces conventional pooling operations with discrete wavelet transform (DWT) to perform multi-resolution analysis. By extracting low-frequency components, DWT improves feature abstraction, enhances structural contrast, and highlights the salience of leakage regions. Multiscale feature storage combined with skip connections ensures effective fusion of local and global information, while inverse wavelet transform is applied during up-sampling to reconstruct high-resolution details without discarding low-frequency information. Together, these strategies significantly improve segmentation performance, as validated by experimental results on hospital datasets.

**Figure 9.**
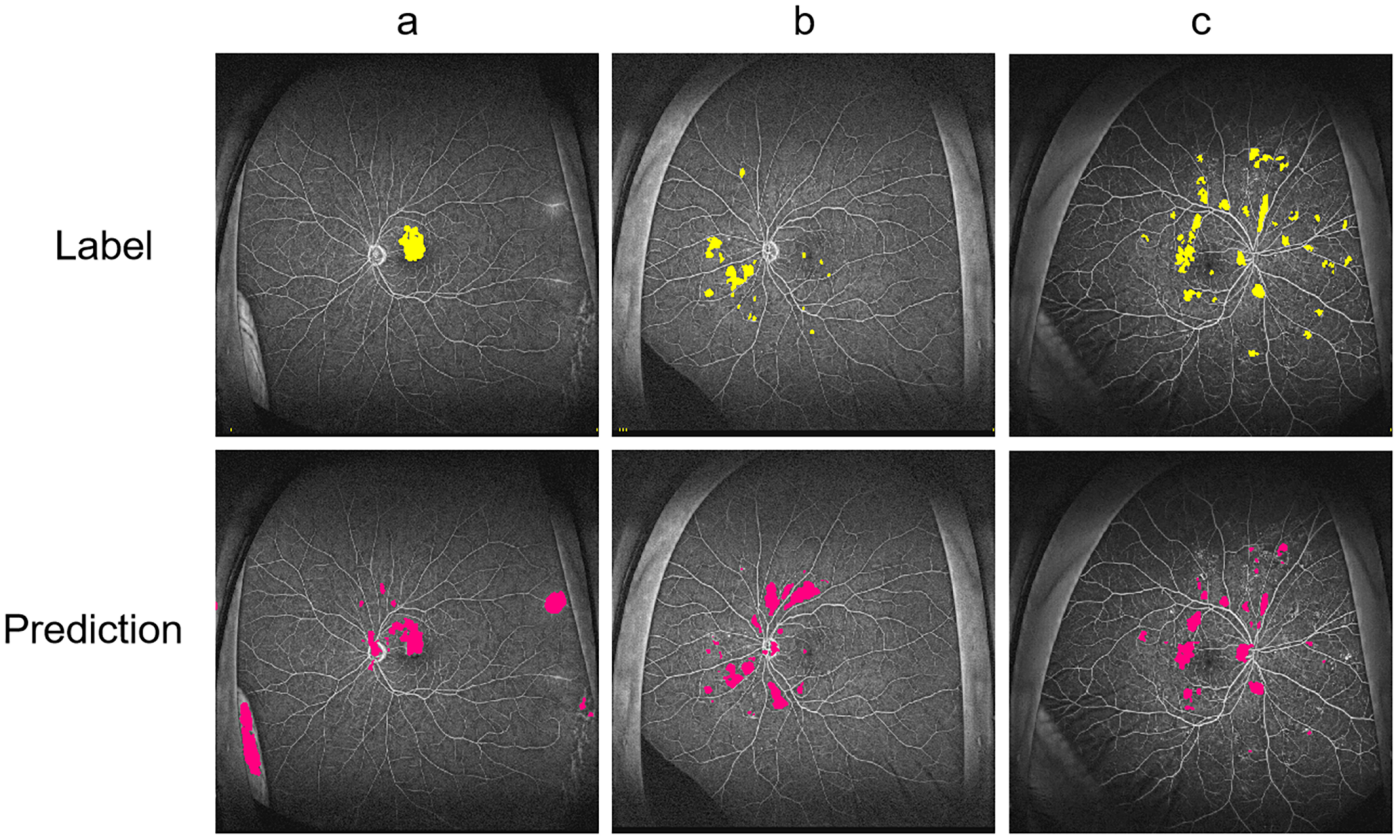
Examples of segmentation errors. (**a**) Misclassification of peripheral hyperfluorescent artifacts as leakage. (**b**) Over-segmentation of vessel-adjacent leakage and false positives from background reflections. (**c**) Fragmented prediction of elongated leakage and missed detection of small low-contrast lesions.

Despite the strong performance of the proposed model, several challenges remain for clinical implementation. First, the degree of automation is limited. While the model provides automatic lesion segmentation, manual correction is often required, particularly in cases where peripheral artifacts are misidentified as pathological leakages. As illustrated in Figure 9, typical failure modes include: (a) misclassification of peripheral hyperfluorescent artifacts as leakages; (b) overestimation and elongation of leakages along major vessels, with background reflections incorrectly segmented as lesions; and (c) fragmented prediction of elongated leakages and missed detection of small, low-contrast foci dispersed in the posterior pole and mid-periphery. These examples highlight the model's sensitivity to non-pathological features and subtle lesion patterns, underscoring the need for greater robustness. Future extensions could incorporate active learning or uncertainty-based correction to reduce human input and improve usability. Second, the model is not yet integrated into clinical decision-making workflows. Although it achieves precise segmentation, it does not currently support diagnostic or treatment planning tasks. Moreover, performance may degrade in complex anatomical regions, such as near large vessels or in subtle leakage patterns where the boundary between pathology and background noise is ambiguous. Multimodal integration with OCT, OCTA, or biomarker data could enhance predictive value and enable more personalized care. Finally, system compatibility presents a practical barrier. Clinical deployment requires Digital Imaging and Communications in Medicine compliant tools with seamless integration into Picture Archiving and Communication System for real-time processing, automated reporting, and interoperability with existing infrastructure. Addressing limitations in robustness to artifacts, clinical workflow integration, and system compatibility will be essential for translating the proposed framework into routine clinical practice.
